# Supplementary material for: Validation of a Multiplex Molecular Macroarray for the Determination of Allergen-Specific IgE Sensitizations in Dogs
Source: Vet Sci. 2024 Oct 7;11(10):482. doi: 10.3390/vetsci11100482 (PMC11512340; doi:10.3390/vetsci11100482)
Supplement: Supplementary file 1 [file vetsci-11-00482-s001.zip › vetsci-3171482-supplementary.pdf]

Table S1: List of allergens - PAX 22.2

Component (C) or extract (E)	Common name	Scientific name	Allergen	Biochemical designation	CCD?
**Grass Pollens**					
E	Bermuda grass	*Cynodon dactylon*	Cyn d		CCD+
C	Bermuda grass	*Cynodon dactylon*	rCyn d 1	Beta-Expansin	
E	Cocksfoot, Orchard grass	*Dactylis glomerata*	Dac g		CCD+
E	Meadow fescue	*Festuca pratensis*	Fes p		CCD+
C	Perennial ryegrass	*Lolium perenne*	rLol p 1	Beta-Expansin	
E	Bahia grass	*Paspalum notatum*	Pas n		CCD+
C	Timothy	*Phleum pratense*	rPhl p 1	Beta-Expansin	
C	Timothy	*Phleum pratense*	rPhl p 2	Expansin	
C	Timothy	*Phleum pratense*	rPhl p 5.0101	Grass group 5/6	
C	Timothy	*Phleum pratense*	rPhl p 6	Grass group 5/6	
C	Timothy	*Phleum pratense*	rPhl p 7	Polcalcin	
C	Timothy	*Phleum pratense*	rPhl p 12	Profilin	
E	Kentucky blue grass	*Poa pratensis*	Poa p		CCD+
E	Ryegrass, cultivated	*Secale cereale*	Sec c_pollen		CCD+
E	Johnson grass	*Sorghum halepense*	Sor h		CCD+
**Tree Pollens**					
E	Acacia	*Acacia mimosa*	Aca m		CCD+
E	Alder	*Alnus glutinosa*	Aln g		CCD+
C	Alder	*Alnus glutinosa*	rAln g 1	PR-10	
C	Alder	*Alnus glutinosa*	rAln g 4	Polcalcin	
E	Silver birch	*Betula verrucosa*	Bet v		CCD+
C	Silver birch	*Betula verrucosa*	rBet v 1	PR-10	
C	Silver birch	*Betula verrucosa*	rBet v 2	Profilin	
C	Silver birch	*Betula verrucosa*	rBet v 6	Isoflavon reductase	
E	Hazel	*Corylus avellana*	Cor a_pollen		CCD+
C	Hazel	*Corylus avellana*	rCor a 1.0103	PR-10	
C	Japanese cedar	*Cryptomeria japonica*	nCry j 1	Pectate lyase	CCD+
C	Arizona cypress	*Cupressus arizonica*	nCup a 1	Pectate lyase	CCD+
E	Cypress	*Cupressus sempervirens*	Cup s		CCD+
C	Beech	*Fagus sylvatica*	rFag s 1	PR-10	
E	Ash	*Fraxinus excelsior*	Fra e		CCD+
C	Ash	*Fraxinus excelsior*	rFra e 1	Ole e 1-family	
E	Walnut	*Juglans regia*	Jug r_pollen		CCD+
EM	Mountain cedar / red cedar	*Juniperus ashei / virginiana*	Jun a_Jun v		CCD+
E	Privet	*Ligustrum vulgare*	Lig v		CCD+
E	Mulberry tree	*Morus rubra*	Mor r		CCD+
E	Olive tree	*Olea Europaea*	Ole e		CCD+
C	Olive	*Olea Europaea*	rOle e 1	Ole e 1-family	
C	Olive	*Olea Europaea*	rOle e 7	nonspecific lipid-transfer protein	
C	Olive	*Olea Europaea*	rOle e 9	1,3 β-glucanase	
C	London plane tree	*Platanus acerifolia*	rPla a 1	Plant invertase	
C	London plane tree	*Platanus acerifolia*	nPla a 2	Polygalacturonase	CCD+
C	London plane tree	*Platanus acerifolia*	rPla a 3	nonspecific lipid-transfer protein	
E	Cottonwood	*Populus nigra*	Pop n		CCD+
E	Elm	*Ulmus campestris*	Ulm c		CCD+
**Weed Pollens**					
E	Pigweed	*Amaranthus retroflexus*	Ama r		CCD+
E	Ragweed	*Ambrosia artemisiifolia*	Amb a		CCD+
C	Ragweed	*Ambrosia artemisiifolia*	rAmb a 1	Pectate lyase	
C	Ragweed	*Ambrosia artemisiifolia*	rAmb a 4	Plant defensin	
E	Mugwort	*Artemisia vulgaris*	Art v		CCD+
C	Mugwort	*Artemisia vulgaris*	rArt v 1.0101	Plant defensin	
C	Mugwort	*Artemisia vulgaris*	rArt v 3.0201	nonspecific lipid-transfer protein	
E	Lamb's quarter	*Chenopodium album*	Che a		CCD+
C	Lamb's quarter	*Chenopodium album*	rChe a 1	Ole e 1-family	
C	Annual mercury	*Mercurialis annua*	rMer a 1	profilin	
E	Wall pellitory	*Parietaria judaica*	Par j		CCD+
C	Wall pellitory	*Parietaria judaica*	rPar j 2	nonspecific lipid-transfer protein	
E	Ribwort / Plantain	*Plantago lanceolata*	Pla l		CCD+
C	Ribwort / Plantain	*Plantago lanceolata*	rPla l 1	Ole e 1-family	
EM	Dock/Sorrel	*Rumex acetosella / crispus*	Rum a_Rum c		CCD+
E	Russian thistle	*Salsola kali*	Sal k		CCD+
C	Russian thistle	*Salsola kali*	rSal k 1	Pectin methylesterase	
E	Nettle	*Urtica dioica*	Urt d		CCD+
**Danders & Epithelia**					
C	Cattle	*Bos domesticus*	rBos d 2	Lipocalin	
C	Dog	*Canis familiaris*	rCan f 1	Lipocalin	
C	Dog	*Canis familiaris*	rCan f 2	Lipocalin	
C	Dog	*Canis familiaris*	nCan f 3	Serum albumin	
C	Dog	*Canis familiaris*	rCan f 4	Lipocalin	
C	Dog	*Canis familiaris*	rCan f 6	Lipocalin	
E	Dog	*Canis familiaris*	Can f_maleurine (including Can f 5)		
C	Dog	*Canis familiaris*	rCan f Fel d 1 like	Fel d 1 like	
C	Guinea pig	*Cavia porcellus*	rCav p 1	Lipocalin	
C	Horse	*Equus caballus*	rEqu c 1	Lipocalin	
C	Horse	*Equus caballus*	nEqu c 3	Serum albumin	
C	Horse	*Equus caballus*	rEqu c 4	Latherin	
C	Cat	*Felis domesticus*	rFel d 1	Uteroglobin	
C	Cat	*Felis domesticus*	nFel d 2	Serum albumin	
C	Cat	*Felis domesticus*	rFel d 4	Lipocalin	
C	Cat	*Felis domesticus*	rFel d 7	Lipocalin	
C	Mouse	*Mus musculus*	rMus m 1	Lipocalin	
C	Rabbit	*Oryctolagus cuniculus*	rOry c 1	Lipocalin	
C	Rabbit	*Oryctolagus cuniculus*	rOry c 2	Lipocalin	
C	Rabbit	*Oryctolagus cuniculus*	rOry c 3	Secretoglobin	
**Mites, Flea and Cockroaches**					
E	*Acarus siro*	*Acarus siro*	Aca s		
C	German cockroach	*Blatella germanica*	rBla g 1	Cockroach group 1	

C	German cockroach	*Blatella germanica*	rBla g 2	Aspartyl protease	
C	German cockroach	*Blatella germanica*	rBla g 4	Calycin	
C	German cockroach	*Blatella germanica*	rBla g 5	Glutathione S-transferase	
C	German cockroach	*Blatella germanica*	rBla g 9	Arginine kinase	
E	*Blomia tropicalis*	*Blomia tropicalis*	Blo t		
C	*Blomia tropicalis*	*Blomia tropicalis*	rBlo t 5	Mite, Group 5	
C	*Blomia tropicalis*	*Blomia tropicalis*	rBlo t 10	Tropomyosin	
C	*Blomia tropicalis*	*Blomia tropicalis*	rBlo t 21	Unknown	
C	Cat flea	*Ctenocephalides felis*	rCte f 1	Unknown	
E	American house dust mite	*Dermatophagoides farinae*	Der f		
C	American house dust mite	*Dermatophagoides farinae*	rDer f 1	Cysteine protease	
C	American house dust mite	*Dermatophagoides farinae*	rDer f 2	NPC2 family	
C	American house dust mite	*Dermatophagoides farinae*	rDer f 15	Chitinase	
C	American house dust mite	*Dermatophagoides farinae*	rDer f 18	Chitin-binding protein	
E	European house dust mite	*Dermatophagoides pteronyssinus*	Der p		
C	European house dust mite	*Dermatophagoides pteronyssinus*	rDer p 1	Cysteine protease	
C	European house dust mite	*Dermatophagoides pteronyssinus*	rDer p 2	NPC2 family	
C	European house dust mite	*Dermatophagoides pteronyssinus*	rDer p 5	Unknown	
C	European house dust mite	*Dermatophagoides pteronyssinus*	rDer p 7	Mite group 7	
C	European house dust mite	*Dermatophagoides pteronyssinus*	rDer p 10	Tropomyosin	
C	European house dust mite	*Dermatophagoides pteronyssinus*	rDer p 11	Myosin, heavy chain	
C	European house dust mite	*Dermatophagoides pteronyssinus*	rDer p 20	Arginine kinase	
C	European house dust mite	*Dermatophagoides pteronyssinus*	rDer p 21	Unknown	
C	European house dust mite	*Dermatophagoides pteronyssinus*	rDer p 23	Peritrophin-like protein domain	
C	*Glycyphagus domesticus*	*Glycyphagus domesticus*	rGly d 2	NPC2 family	
E	*Lepidoglyphus destructor*	*Lepidoglyphus destructor*	Lep d		
C	*Lepidoglyphus destructor*	*Lepidoglyphus destructor*	rLep d 2	NPC2 family	
C	American cockroach	*Periplaneta americana*	Per a 6	Troponin C	
C	American cockroach	*Periplaneta americana*	rPer a 7	Tropomyosin	
E	*Tyrophagus putrescentiae*	*Tyrophagus putrescentiae*	Tyr p		
C	*Tyrophagus putrescentiae*	*Tyrophagus putrescentiae*	rTyr p 2	NPC2 family	
**Moulds & Yeasts**					
E	*Alternaria alternata*	*Alternaria alternata*	Alt a		
C	*Alternaria alternata*	*Alternaria alternata*	rAlt a 1	Alt a 1-family	
C	*Alternaria alternata*	*Alternaria alternata*	rAlt a 6	Enolase	
E	*Aspergillus fumigatus*	*Aspergillus fumigatus*	Asp f		
C	*Aspergillus fumigatus*	*Aspergillus fumigatus*	rAsp f 1	Mitogillin family	
C	*Aspergillus fumigatus*	*Aspergillus fumigatus*	rAsp f 3	Peroxysomal protein	
C	*Aspergillus fumigatus*	*Aspergillus fumigatus*	rAsp f 4	Unknown	
C	*Aspergillus fumigatus*	*Aspergillus fumigatus*	rAsp f 6	Mn superoxid-dismutase	
E	*Cladosporium herbarum*	*Cladosporium herbarum*	Cla h		
C	*Cladosporium herbarum*	*Cladosporium herbarum*	rCla h 8	Short-chain dehydrogenase	
E	*Penicilium chrysogenum*	*Penicilium chrysogenum*	Pen ch		
E	*Malassezia pachydermatis*	*Malassezia pachydermatis*	Mala p		
C	*Malassezia sympodialis*	*Malassezia sympodialis*	rMala s 1	Peroxysomal protein	
C	*Malassezia sympodialis*	*Malassezia sympodialis*	rMala s 5	Unknown	
C	*Malassezia sympodialis*	*Malassezia sympodialis*	rMala s 6	Cyclophilin	
C	*Malassezia sympodialis*	*Malassezia sympodialis*	rMala s 9	Unknown	
C	*Malassezia sympodialis*	*Malassezia sympodialis*	rMala s 11	Mn superoxid-dismutase	
**Insect Venoms**					
E	Honey bee venom	*Apis mellifera*	Api m		insect CCD+
C	Honey bee venom	*Apis mellifera*	nApi m 1	Phospholipase A2	insect CCD+
C	Honey bee venom	*Apis mellifera*	rApi m 2	Hyaluronidase	
C	Honey bee venom	*Apis mellifera*	rApi m 3	Acid phosphatase	
C	Honey bee venom	*Apis mellifera*	rApi m 5	Dipeptidylpeptidase IV	
C	Honey bee venom	*Apis mellifera*	rApi m 10	Icarapin variant 2	
E	Long-headed wasp venom	*Dolichovespula spp.*	Dol spp		insect CCD+
E	Paper wasp venom	*Polistes dominulus*	Pol d		insect CCD+
C	Paper wasp venom	*Polistes dominulus*	rPol d 5	Antigen 5	
E	Fire ant venom	*Solenopsis richteri & S. invicta*	Sol spp		insect CCD+
E	Common wasp venom	*Vespula vulgaris*	Ves v		insect CCD+
C	Common wasp venom	*Vespula vulgaris*	rVes v 1	Phospholipase A1	
C	Common wasp venom	*Vespula vulgaris*	rVes v 5	Antigen 5	
**Cereals & Seeds**					
E	Oat	*Avena sativa*	Ave s		CCD+
E	Buckwheat	*Fagopyrum esculentum*	Fag e		CCD+
C	Buckwheat	*Fagopyrum esculentum*	nFag e 2	2S albumin	CCD+
E	Sunflower seed	*Helianthus annuus*	Hel a		CCD+
E	Barley	*Hordeum vulgare*	Hor v		CCD+
E	Rice	*Oryza sativa*	Ory s		CCD+
C	Rice	*Oryza sativa*	nOry s_GLUB1	Glutelin B1	CCD+
E	Millet	*Panicum miliaceum*	Pan m		CCD+
E	Rye, cultivated	*Secale cereale*	Sec c_flour		CCD+
E	Wheat	*Triticum aestivum*	Tri a		CCD+
C	Wheat	*Triticum aestivum*	rTri a 14	nonspecific lipid-transfer protein	
C	Wheat	*Triticum aestivum*	rTri a 19	Omega-5-gliadin	
C	Wheat	*Triticum aestivum*	nTri a aA_TI	Alpha-amylase trypsin-inhibitor	
E	Corn, cereal	*Zea mays*	Zea m		CCD+
C	Corn, cereal	*Zea mays*	rZea m 14	nonspecific lipid-transfer protein	
C	Corn, cereal	*Zea mays*	rZea m_GBSSI	Granule-bound starch synthase 1	
**Fruits**					
C	Apple	*Malus domestica*	rMal d 1	PR-10	
C	Apple	*Malus domestica*	rMal d 2	Thaumatin-like protein	
C	Apple	*Malus domestica*	rMal d 3	nonspecific lipid-transfer protein	
**Legumes & Nuts**					
C	Peanut	*Arachis hypogea*	nAra h 1	7/8S globulin	CCD+
C	Peanut	*Arachis hypogea*	rAra h 2	2S albumin	
C	Peanut	*Arachis hypogea*	nAra h 3	11S globulin	
C	Peanut	*Arachis hypogea*	rAra h 5	Profilin	
C	Peanut	*Arachis hypogea*	rAra h 6	2S albumin	
C	Peanut	*Arachis hypogea*	rAra h 8	PR-10	
C	Peanut	*Arachis hypogea*	rAra h 9	nonspecific lipid-transfer protein	

C	Peanut	*Arachis hypogea*	rAra h 15	Oleosin	
E	Soy	*Glycine max*	Gly m		CCD+
C	Soy	*Glycine max*	rGly m 4	PR-10	
C	Soy	*Glycine max*	nGly m 5	7/8S globulin	CCD+
C	Soy	*Glycine max*	nGly m 6	11S globulin	CCD+
C	Soy	*Glycine max*	rGly m 8	2S albumin	
E	Lentil	*Lens culinaris*	Len c		CCD+
C	Lentil	*Lens culinaris*	rLen c 1	7/8S globulin	
C	Lentil	*Lens culinaris*	nLen c 2	Seed-specific biotinylated protein	
C	Lentil	*Lens culinaris*	rLen c 3	nonspecific lipid-transfer protein	
E	Pea	*Pisum sativum*	Pis s		CCD+
C	Pea	*Pisum sativum*	rPis s 1	7/8S globulin	
C	Pea	*Pisum sativum*	rPis s 2	7/8S globulin	
C	Pea	*Pisum sativum*	rPis s 3	nonspecific lipid-transfer protein	
**Egg & Milk**					
E	Cow's milk	*Bos domesticus*	Bos d_milk		
C	Cow's milk	*Bos domesticus*	nBos d 4	α-lactalbumin	
C	Cow's milk	*Bos domesticus*	nBos d 5	β-lactoglobulin	
C	Cow's milk	*Bos domesticus*	nBos d 8	Casein	
E	Egg white	*Gallus domesticus*	Gal d_white		
E	Egg yolk	*Gallus domesticus*	Gal d_yolk		
C	Egg white	*Gallus domesticus*	nGal d 1	Ovomucoid	
C	Egg white	*Gallus domesticus*	nGal d 2	Ovalbumin	
C	Egg white	*Gallus domesticus*	nGal d 3	Ovotransferrin	
C	Egg white	*Gallus domesticus*	nGal d 4	Lysozym C	
C	Egg yolk	*Gallus domesticus*	nGal d 5	Serum albumin	
**Meats**					
E	Beef	*Bos domesticus*	Bos d_meat		
C	Beef	*Bos domesticus*	nBos d 6	Serum albumin	
C	Beef	*Bos domesticus*	nBos d 7	Immunoglobulin G	
C	Beef	*Bos domesticus*	nBos d_ACTA1	Alpha actin	
C	Beef	*Bos domesticus*	nBos d_LDHA	Lactate dehydrogenase A	
E	Horse	*Equus caballus*	Equ c_meat		
E	Rabbit	*Oryctolagus spp.*	Ory_meat		
C	Rabbit	*Oryctolagus cuniculus*	nOry c_CKM	Creatine kinase M	
C	Rabbit	*Oryctolagus cuniculus*	nOry c_GAPDH	GAPDH	
C	Rabbit	*Oryctolagus cuniculus*	nOry c_PGM1	Phosphoglucomutase-1	
C	Rabbit	*Oryctolagus cuniculus*	nOry c_PKM	Pyruvate kinase M	
C	Rabbit	*Oryctolagus cuniculus*	nOry c_TPI1	Triose phosphate isomerase 1	
E	Lamb	*Ovis aries*	Ovi a_meat		
C	Lamb	*Ovis aries*	nOvi a_IgG	Immunoglobulin G	
E	Pig	*Sus domesticus*	Sus d_meat		
C	Pig	*Sus domesticus*	rSus d 1	Serum albumin	
E	Chicken	*Gallus domesticus*	Gal d_meat		
C	Chicken	*Gallus domesticus*	rGal d 7	Myosin light chain	
C	Chicken	*Gallus domesticus*	rGal d 9	Beta-enolase (enolase -3)	
C	Chicken	*Gallus domesticus*	Gal d_PKM	Pyruvate kinase M	
E	Turkey	*Meleagris gallopavo*	Mel g		
E	Mealworm	*Tenebrio molitor*	Ten m		
**Fish**					
E	Herring, Atlantic	*Clupea harengus*	Clu h		
C	Herring, Atlantic	*Clupea harengus*	rClu h 1	β-parvalbumin	
E	Cod, Atlantic	*Gadus morhua*	Gad m		
C	Cod, Atlantic	*Gadus morhua*	nGad m 1	β-parvalbumin	
CM	Cod, Atlantic	*Gadus morhua*	nGad m 2+3	Aldolase/beta-enolase (enolase -3)	
C	Cod, Atlantic	*Gadus morhua*	nGad m 4	Tropomyosin	
E	Salmon, Atlantic	*Salmo salar*	Sal s		
C	Salmon, Atlantic	*Salmo salar*	rSal s 1	β-parvalbumin	
C	Salmon, Atlantic	*Salmo salar*	rSal s 2	Beta-enolase (enolase -3)	
C	Salmon, Atlantic	*Salmo salar*	rSal s 3	Aldolase	
C	Salmon, Atlantic	*Salmo salar*	rSal s 4	Tropomyosin	
C	Salmon, Atlantic	*Salmo salar*	nSal s 6	Collagen	
C	Salmon, Atlantic	*Salmo salar*	rSal s 7	Creatine kinase M	
C	Salmon, Atlantic	*Salmo salar*	rSal s 8	Triosephosphate isomerase 1	
E	Mackerel, Atlantic	*Scomber scombrus*	Sco s		
C	Mackerel, Atlantic	*Scomber scombrus*	rSco s 1	β-parvalbumin	
E	Tuna	*Thunnus albacares*	Thu a		
C	Tuna	*Thunnus albacares*	rThu a 1	β-parvalbumin	
**Tubers**					
E	Carrot	*Daucus carota*	Dau c		CCD+
C	Carrot	*Daucus carota*	rDau c 1	PR-10	
E	Tomato	*Solanum lycopersicum*	Sola l		CCD+
C	Tomato	*Solanum lycopersicum*	rSola l 6	nonspecific lipid-transfer protein	
E	Potato	*Solanum tuberosum*	Sola t		CCD+
C	Potato	*Solanum tuberosum*	rSola t 2	Aspartic protease inhibitor	
C	Potato	*Solanum tuberosum*	rSola t_GBSSI	Granule-bound starch synthase 1	
**Controls**					
C	Hom s lactoferrin	*rice origin*	rHom s LF	CCD	CCD+
C	Hom s lactoferrin	*human origin*	Hom s LF_wo	without CCD	
C	Hom s_albumin	*Homo sapiens*	Hom s_SA_wo	Serum albumin without CCD	
C	Hom s_albumin_CCD	*Homo sapiens*	Hom s_SA_CCD	Serum albumin with CCD	CCD+

ALTOGETHER	
**# Extracts (E)**	**75**
**# Extract Mixes (EM)**	**2**
**# Components (C)**	**169**
**# Component Mixes (CM)**	**1**
**TOTAL**	**247**
**% of components**	**69%**

Table S2: seropositivity rates 2023

Rank	E/C	Common name	Scientific name	Allergen	Biochemical designation	% positivity
1	E	American house dust mite	*Dermatophagoides farinae*	**Der f**		60,83%
2	C	Honey bee venom	*Apis mellifera*	**Api m 1**	Phospholipase A2	22,81%
3	E	Honey bee venom	*Apis mellifera*	**Api m**		20,58%
4	C	Common wasp venom	*Vespula vulgaris*	**Ves v 5**	Antigen 5	17,37%
5	C	Honey bee venom	*Apis mellifera*	**Api m 3**	Acid phosphatase	16,84%
6	C	*Tyrophagus putrescentiae*	*Tyrophagus putrescentiae*	**Tyr p 2**	NPC2 family	16,64%
7	C	Common wasp venom	*Vespula vulgaris*	**Ves v 1**	Phospholipase A1	16,28%
8	E	*Acarus siro*	*Acarus siro*	**Aca s**		13,88%
9	E	*Tyrophagus putrescentiae*	*Tyrophagus putrescentiae*	**Tyr p**		12,43%
10	C	Honey bee venom	*Apis mellifera*	**Api m 10**	Icarapin variant 2	11,82%
11	C	Honey bee venom	*Apis mellifera*	**Api m 2**	Hyaluronidase	11,70%
12	E	Common wasp venom	*Vespula vulgaris*	**Ves v**		10,97%
13	E	Ragweed	*Ambrosia artemisiifolia*	**Amb a**		9,95%
14	C	Cat flea	*Ctenocephalides felis*	**Cte f 1**	unknown	9,02%
15	C	Wall pellitory	*Parietaria judaica*	**Par j 2**	nsLTP	8,61%
16	E	Wall pellitory	*Parietaria judaica*	**Par j**		8,32%
17	E	Russian thistle	*Salsola kali*	**Sal k**		8,25%
18	E	Cypress	*Cupressus sempervirens*	**Cup s**		7,63%
19	C	Beech	*Fagus sylvatica*	**Fag s 1**	PR-10	7,37%
20	E	*Blomia tropicalis*	*Blomia tropicalis*	**Blo t**		6,61%
21	E	European house dust mite	*Dermatophagoides pteronyssinus*	**Der p**		5,86%
22	C	American house dust mite	*Dermatophagoides farinae*	**Der f 2**	NPC2 family	5,38%
23	C	Paper wasp venom	*Polistes dominulus*	**Pol d 5**	Antigen 5	5,03%
24	C	*Glycyphagus domesticus*	*Glycyphagus domesticus*	**Gly d 2**	NPC2 family	5,00%
25	C	American house dust mite	*Dermatophagoides farinae*	**Der f 1**	Cysteine protease	4,90%
26	C	European house dust mite	*Dermatophagoides pteronyssinus*	**Der p 2**	NPC2 family	4,69%
27	C	American cockroach	*Periplaneta americana*	**Per a 7**	Tropomyosin	4,57%
28	C	Blomia tropicalis	*Blomia tropicalis*	**Blo t 10**	Tropomyosin	4,55%
29	EM	Mountain cedar / red cedar	*Juniperus ashei / virginiana*	**Jun a_Jun v**		4,38%
30	E	*Lepidoglyphus destructor*	*Lepidoglyphus destructor*	**Lep d**		4,22%
31	E	Bahia grass	*Paspalum notatum*	**Pas n**		4,10%
32	C	Arizona cypress	*Cupressus arizonica*	**Cup a 1**	Pectate lyase	3,68%
33	C	*Malassezia sympodialis*	*Malassezia sympodialis*	**Mala s 1**		3,68%
34	E	Ryegrass, cultivated	*Secale cereale*	**Sec c_pollen**		3,49%
35	C	Japanese cedar	*Cryptomeria japonica*	**Cry j 1**	Pectate lyase	3,11%
36	EM	Dock/Sorrel	*Rumex acetosella / crispus*	**Rum a_Rum c**		3,09%
37	E	Long-headed wasp venom	*Dolichovespula spp.*	**Dol spp**		2,93%
38	E	Walnut	*Juglans regia*	**Jug r_pollen**		2,89%
39	C	European house dust mite	*Dermatophagoides pteronyssinus*	**Der p 1**	Cysteine protease	2,64%
40	C	European house dust mite	*Dermatophagoides pteronyssinus*	**Der p 11**	Myosin, heavy chain	2,63%
41	C	Ribwort / Plantain	*Plantago lanceolata*	**Pla l 1**	Ole e 1-family	2,61%
42	C	European house dust mite	*Dermatophagoides pteronyssinus*	**Der p 10**	Tropomyosin	2,39%
43	E	Meadow fescue	*Festuca pratensis*	**Fes p**		2,38%
44	C	European house dust mite	*Dermatophagoides pteronyssinus*	**Der p 21**	Unknown	2,38%
45	C	Olive	*Olea Europaea*	**Ole e 1**	Ole e 1-family	2,28%
46	E	Cocksfoot, Orchard grass	*Dactylis glomerata*	**Dac g**		2,13%
47	E	Acacia	*Acacia mimosa*	**Aca m**		2,04%
48	C	*Cladosporium herbarum*	*Cladosporium herbarum*	**Cla h 8**	Short-chain dehydrogenase	2,03%
49	C	*Aspergillus fumigatus*	*Aspergillus fumigatus*	**Asp f 6**	Mn superoxid-dismutase	2,02%
50	E	Ribwort / Plantain	*Plantago lanceolata*	**Pla l**		1,91%
51	C	Russian thistle	*Salsola kali*	**Sal k 1**	Pectin methylesterase	1,91%
52	E	Pigweed	*Amaranthus retroflexus*	**Ama r**		1,90%
53	C	London plane tree	*Platanus acerifolia*	**Pla a 2**	Polygalacturonase	1,87%
54	C	Ragweed	*Ambrosia artemisiifolia*	**Amb a 4**	Plant defensin	1,83%
55	C	Alder	*Alnus glutinosa*	**Aln g 1**	PR-10	1,80%
56	C	*Malassezia sympodialis*	*Malassezia sympodialis*	**Mala s 11**	Mn superoxid-dismutase	1,75%
57	C	European house dust mite	*Dermatophagoides pteronyssinus*	**Der p 5**	Unknown	1,75%
58	E	Kentucky blue grass	*Poa pratensis*	**Poa p**		1,74%
59	C	Blomia tropicalis	*Blomia tropicalis*	**Blo t 5**	Mite, Group 5	1,59%
60	C	Cat	*Felis domesticus*	**Fel d 2**	Serum albumin	1,58%
61	C	Timothy	*Phleum pratense*	**Phl p 6**	Grass group 5/6	1,45%
62	C	Timothy	*Phleum pratense*	**Phl p 1**	Beta-Expansin	1,42%
63	C	*Aspergillus fumigatus*	*Aspergillus fumigatus*	**Asp f 1**	Mitogillin family	1,41%
64	E	Cottonwood	*Populus nigra*	**Pop n**		1,36%
65	C	Timothy	*Phleum pratense*	**Phl p 2**	Expansin	1,35%
66	C	*Malassezia sympodialis*	*Malassezia sympodialis*	**Mala s 5**	Unknown	1,32%
67	C	Silver birch	*Betula verrucosa*	**Bet v 1**	PR-10	1,31%
68	C	Silver birch	*Betula verrucosa*	**Bet v 6**	Isoflavon reductase	1,28%
69	C	European house dust mite	*Dermatophagoides pteronyssinus*	**Der p 20**	Arginine kinase	1,21%
70	C	*Malassezia sympodialis*	*Malassezia sympodialis*	**Mala s 9**	Unknown	1,20%
71	C	Perennial ryegrass	*Lolium perenne*	**Lol p 1**	Beta-Expansin	1,17%
72	C	Bermuda grass	*Cynodon dactylon*	**Cyn d 1**	Beta-Expansin	1,16%
73	E	Alder	*Alnus glutinosa*	**Aln g**		1,11%
74	C	Mugwort	*Artemisia vulgaris*	**Art v 1**	Plant defensin	1,11%
75	C	European house dust mite	*Dermatophagoides pteronyssinus*	**Der p 23**	Peritrophin-like protein domain	1,10%
76	C	*Aspergillus fumigatus*	*Aspergillus fumigatus*	**Asp f 3**	Peroxysomal protein	1,07%
77	C	Cat	*Felis domesticus*	**Fel d 1**	Uteroglobin	1,07%
78	C	*Malassezia sympodialis*	*Malassezia sympodialis*	**Mala s 6**	Cyclophilin	1,04%
79	E	Johnson grass	*Sorghum halepense*	**Sor h**		1,00%
80	C	Timothy	*Phleum pratense*	**Phl p 5.0101**	Grass group 5/6	0,98%
81	E	Privet	*Ligustrum vulgare*	**Lig v**		0,97%
82	C	Rabbit	*Oryctolagus cuniculus*	**Ory c 2**	Lipocalin	0,88%
83	C	Dog	*Canis familiaris*	**Can f 4**	Lipocalin	0,88%
84	C	German cockroach	*Blatella germanica*	**Bla g 5**	Glutathione S-transferase	0,86%
85	C	*Alternaria alternata*	*Alternaria alternata*	**Alt a 1**	Alt a 1-family	0,86%
86	E	*Malassezia pachydermatis*	*Malassezia pachydermatis*	**Mala p**		0,84%

Legend
grass pollens
tree pollens
weed pollens
mites & insects
fungi
epithelia
insect venoms

**87**	C	Horse	*Equus caballus*	**Equ c 4**	Latherin	0,80%
**88**	E	Mugwort	*Artemisia vulgaris*	**Art v**		0,78%
**89**	C	Timothy	*Phleum pratense*	**Phl p 7**	Polcalcin	0,76%
**90**	C	Hazel	*Corylus avellana*	**Cor a 1.0103**	PR-10	0,76%
**91**	C	Alder	*Alnus glutinosa*	**Aln g 4**	Polcalcin	0,74%
**92**	C	Lamb's quarter	*Chenopodium album*	**Che a 1**	Ole e 1-family	0,74%
**93**	E	Lamb's quarter	*Chenopodium album*	**Che a**		0,73%
**94**	C	*Lepidoglyphus destructor*	*Lepidoglyphus destructor*	**Lep d 2**	NPC2 family	0,68%
**95**	C	Ash	*Fraxinus excelsior*	**Fra e 1**	Ole e 1-family	0,64%
**96**	C	American house dust mite	*Dermatophagoides farinae*	**Der f 18**	Chitin-binding protein	0,62%
**97**	C	*Aspergillus fumigatus*	*Aspergillus fumigatus*	**Asp f 4**	Unknown	0,62%
**98**	E	Hazel	*Corylus avellana*	**Cor a_pollen**		0,61%
**99**	C	American house dust mite	*Dermatophagoides farinae*	**Der f 15**	Chitinase	0,61%
**100**	E	*Alternaria alternata*	*Alternaria alternata*	**Alt a**		0,60%
**101**	C	Olive	*Olea Europaea*	**Ole e 9**	1,3 β-glucanase	0,60%
**102**	E	Silver birch	*Betula verrucosa*	**Bet v**		0,56%
**103**	C	Honey bee venom	*Apis mellifera*	**Api m 5**	Dipeptidylpeptidase IV	0,54%
**104**	C	Mugwort	*Artemisia vulgaris*	**Art v 3**	nsLTP	0,53%
**105**	C	Rabbit	*Oryctolagus cuniculus*	**Ory c 1**	Lipocalin	0,53%
**106**	E	Ash	*Fraxinus excelsior*	**Fra e**		0,52%
**107**	C	German cockroach	*Blatella germanica*	**Bla g 9**	Arginine kinase	0,52%
**108**	C	*Blomia tropicalis*	*Blomia tropicalis*	**Blo t 21**	unknown	0,48%
**109**	C	Ribwort / Plantain	*Plantago lanceolata*	**Pla a 1**	Ole e 1-family	0,47%
**110**	E	*Aspergillus fumigatus*	*Aspergillus fumigatus*	**Asp f**		0,47%
**111**	C	Timothy	*Phleum pratense*	**Phl p 12**	Profilin	0,44%
**112**	E	Elm	*Ulmus campestris*	**Ulm c**		0,43%
**113**	C	German cockroach	*Blatella germanica*	**Bla g 2**	Aspartyl protease	0,42%
**114**	C	Annual mercury	*Mercurialis annua*	**Mer a 1**	profilin	0,41%
**115**	C	Dog	*Canis familiaris*	**Can f 1**	Lipocalin	0,41%
**116**	C	Guinea pig	*Cavia porcellus*	**Cav p 1**	Lipocalin	0,41%
**117**	C	Cat	*Felis domesticus*	**Fel d 4**	Lipocalin	0,39%
**118**	C	Silver birch	*Betula verrucosa*	**Bet v 2**	Profilin	0,37%
**119**	C	Horse	*Equus caballus*	**Equ c 3**	Serum albumin	0,36%
**120**	C	European house dust mite	*Dermatophagoides pteronyssinus*	**Der p 7**	Mite group 7	0,36%
**121**	C	Dog	*Canis familiaris*	**Can f_Fd1**	Fel d 1 like	0,36%
**122**	E	*Cladosporium herbarum*	*Cladosporium herbarum*	**Cla h**		0,33%
**123**	C	German cockroach	*Blatella germanica*	**Bla g 4**	Calycin	0,33%
**124**	C	Annual mercury	*Mercurialis annua*	**Amb a 1**	profilin	0,32%
**125**	C	Horse	*Equus caballus*	**Equ c 1**	Lipocalin	0,32%
**126**	E	Dog	*Canis familiaris*	**Can f_male urine**		0,31%
**127**	C	Rabbit	*Oryctolagus cuniculus*	**Ory c 3**	Secretoglobin	0,29%
**128**	C	*Alternaria alternata*	*Alternaria alternata*	**Alt a 6**	Enolase	0,29%
**129**	E	Bermuda grass	*Cynodon dactylon*	**Cyn d**		0,28%
**130**	E	Mulberry tree	*Morus rubra*	**Mor r**		0,28%
**131**	C	Cat	*Felis domesticus*	**Fel d 7**	Lipocalin	0,28%
**132**	C	London plane tree	*Platanus acerifolia*	**Pla a 3**	nsLTP	0,26%
**133**	C	Dog	*Canis familiaris*	**Can f 3**	Serum albumin	0,25%
**134**	E	Nettle	*Urtica dioica*	**Urt d**		0,24%
**135**	C	American cockroach	*Periplaneta americana*	**Per a 6**	Troponin C	0,23%
**136**	C	Olive	*Olea Europaea*	**Ole e 7**	nsLTP	0,22%
**137**	C	Cattle	*Bos domesticus*	**Bos d 2**	Lipocalin	0,22%
**138**	E	Fire ant venom	*Solenopsis richteri & S. invicta*	**Sol spp**		0,21%
**139**	C	German cockroach	*Blatella germanica*	**Bla g 1**	Cockroach group 1	0,18%
**140**	C	Dog	*Canis familiaris*	**Can f 2**	Lipocalin	0,13%
**141**	C	Dog	*Canis familiaris*	**Can f 6**	Lipocalin	0,08%
**142**	C	Mouse	*Mus musculus*	**Mus m 1**	Lipocalin	0,08%
**143**	E	Paper wasp venom	*Polistes dominulus*	**Pol d**		0,07%
**144**	E	Olive tree	*Olea Europaea*	**Ole e_pollen**		0,05%
**145**	E	*Penicilium chrysogenum*	*Penicilium chrysogenum*	**Pen ch**		0,04%
